# Using a UK Virtual Supermarket to Examine Purchasing Behavior Across Different Income Groups in the United Kingdom: Development and Feasibility Study

**DOI:** 10.2196/jmir.7982

**Published:** 2017-10-09

**Authors:** Anja Mizdrak, Wilma Elzeline Waterlander, Mike Rayner, Peter Scarborough

**Affiliations:** ^1^ Burden of Disease Epidemiology, Equity and Cost-Effectiveness Programme (BODE3) Department of Public Health University of Otago, Wellington Wellington New Zealand; ^2^ National Institute of Health Innovation University of Auckland Auckland New Zealand; ^3^ Centre on Population Approaches for Non-Communicable Disease Prevention Nuffield Department of Population Health University of Oxford Oxford United Kingdom

**Keywords:** food, diet, public health, United Kingdom, socioeconomic status

## Abstract

**Background:**

The majority of food in the United Kingdom is purchased in supermarkets, and therefore, supermarket interventions provide an opportunity to improve diets. Randomized controlled trials are costly, time-consuming, and difficult to conduct in real stores. Alternative approaches of assessing the impact of supermarket interventions on food purchases are needed, especially with respect to assessing differential impacts on population subgroups.

**Objective:**

The aim of this study was to assess the feasibility of using the United Kingdom Virtual Supermarket (UKVS), a three-dimensional (3D) computer simulation of a supermarket, to measure food purchasing behavior across income groups.

**Methods:**

Participants (primary household shoppers in the United Kingdom with computer access) were asked to conduct two shopping tasks using the UKVS and complete questionnaires on demographics, food purchasing habits, and feedback on the UKVS software. Data on recruitment method and rate, completion of study procedure, purchases, and feedback on usability were collected to inform future trial protocols.

**Results:**

A total of 98 participants were recruited, and 46 (47%) fully completed the study procedure. Low-income participants were less likely to complete the study (*P*=.02). Most participants found the UKVS easy to use (38/46, 83%) and reported that UKVS purchases resembled their usual purchases (41/46, 89%).

**Conclusions:**

The UKVS is likely to be a useful tool to examine the effects of nutrition interventions using randomized controlled designs. Feedback was positive from participants who completed the study and did not differ by income group. However, retention was low and needs to be addressed in future studies. This study provides purchasing data to establish sample size requirements for full trials using the UKVS.

## Introduction

### Background

Unhealthy diets pose a substantial threat to public health. Globally, dietary risk factors account for 11.3 million deaths and 241.4 disability-adjusted life years per year [1]. In the United Kingdom, dietary risk factors account for nearly one-fifth of deaths and one-tenth of disability-adjusted life years [2]. Improvements in diet could be achieved by tackling key determinants of food choice.

Price is a key determinant of food choice: 36% of shoppers consider price to be the most important, and 90% of shoppers list price in the top five most important influences on food purchases in the United Kingdom [3]. Health-related food taxes and subsidies (HRFTS) are interventions that raise the price of unhealthy foods or lower the price of healthy foods to encourage healthier diets. Several HRFTS have been implemented. Sugar-sweetened beverage taxes have been introduced in Mexico, France, and Chile [4-6] and recently announced in the United Kingdom [7]. Dominica applies an excise tax to foods and drinks with high sugar content; Hungary has a public health tax that is applied to selected foods, including those with high salt or sugar content; and Finland levies taxes on confectionery and ice-cream [6]. Other HRFTS that have been suggested include subsidies on healthy foods and taxes based on nutrient profiling models [8]. HRFTS are one of the several population interventions recommended by the World Health Organization [9].

In the United Kingdom, the majority of food is purchased in supermarket chains [10]. This makes supermarkets an important environment to consider when examining the impact of specific price changes on food purchasing. However, testing the impact of HRFTS and other interventions in real supermarkets is difficult. Supermarkets may not wish to participate in trials where there is a risk of reduced sales, loss of customers, or negative media coverage (eg, taxes on unhealthy foods). Nationwide promotional and pricing strategies by retailers may limit what interventions can be implemented at individual sites, and there may be reluctance to implement interventions that depend on the input of supermarket staff time (eg, changing product placement). The resources required to run full trials in real supermarkets (eg, the cost of subsidies) also prohibit the number of interventions that can be tested in real supermarkets. Evidence on the effects of interventions on supermarket purchases may therefore need to be gathered by other means—virtual supermarkets are one prospect.

A virtual supermarket is a three-dimensional (3D) graphical representation of a real supermarket in which participants can complete shopping tasks. Virtual supermarkets have been previously used to examine price interventions and have been validated against real supermarket purchases [11-13]. In these virtual supermarkets, participants are asked to complete a shopping task specified by researchers and do not pay real money or receive real versions of the foods purchased in the virtual environment. The New Zealand Virtual Supermarket (NZVS) was validated by comparing participants’ real-life purchases with those made in the NZVS over a 3-week period [13]. The validation study found that shopping patterns in the NZVS were comparable with those in real life: the four food groups with the highest relative expenditure were the same, and there was no trend of overspending in the NZVS.

### Objectives

This paper introduces a United Kingdom Virtual Supermarket (UKVS) that resembles a small supermarket store and presents the results of a feasibility study assessing recruitment, retention, purchasing variability, and participant responses to the newly developed software. In this study, we recruited participants to complete two shopping tasks and sociodemographic questionnaires at a single time point. The shopping tasks asked participants to purchase all foods for their household for at least the next day, which was in line with previous studies [14-16] and likely to be comparable with smaller top-up shops that comprise around 60% of household food spending in the United Kingdom [17]. We also examined differences in the above across different income groups. Lack of evidence on the differential impacts of HRFTS among population subgroups has been identified in a number of reviews [18-20]. In addition, previous experimental studies of food pricing strategies have observed differential recruitment and retention rates by participant group, possibly linked to differences in ease of participation [21]. Finally, as no previous UKVS studies have been done, we needed to collect data on purchases and variability in purchases to assess likely sample sizes for randomized controlled trials (RCTs) in the UKVS.

This study aimed to address the following research questions:

- How effective are online methods, plus snowballing, for the recruitment of participants for a UKVS study?
- What are the dropout rates for a UKVS study?
- How much variability is there in next-day shopping behavior in the UKVS?
- How do participants report ease of participation and appreciation of the UKVS?
- Do recruitment and dropout rates, variability in next-day shopping behavior, and ease of participation vary by income group?

## Methods

### Development of the UK Virtual Supermarket

The use of the existing Dutch Virtual Supermarket [22] as the template for a new UKVS was agreed with researchers at VU University Amsterdam and SURFsara, a not-for-profit software development company that was responsible for the development of both the Dutch and New Zealand versions of the virtual supermarket.

**Figure 1:**
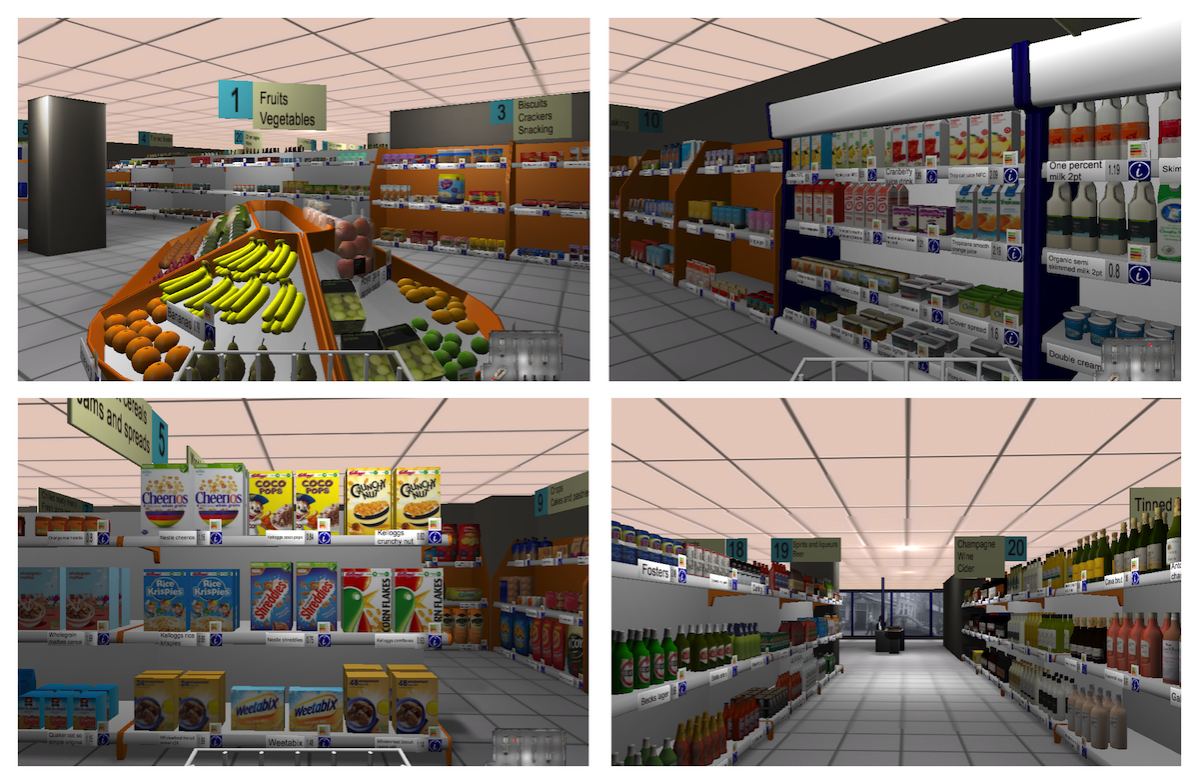
Screenshots from the United Kingdom Virtual Supermarket.

The creation of the UKVS from the Dutch Virtual Supermarket template comprised the replacement of Dutch products with UK products, changes to the software to make it fit within the UK context (eg, English aisle signs), and changes to the study procedure format. The UKVS most closely represents a smaller supermarket in the United Kingdom and not a large superstore. Screenshots from the completed UKVS are displayed in Figure 1.

Shelf spaces were allocated to food categories based on the distribution of shelf spaces in surveys of three actual small supermarket stores in the United Kingdom. This set the number of product spaces that were available in each food category. Data from the Living Costs and Food Survey [23] were used to check that popular food categories were represented in the shelf space allocation. Nonfood items were excluded from the UKVS, and the virtual supermarket did not contain end-of-aisle displays or products at the checkout.

An online supermarket [24] was used to review the full range of products available in each food category allocated spaces in the UKVS. We recorded information on the number of products, price range, sizes available, brands, top sellers, and types of product. Specific products were selected to reflect popularity (from the *top sellers* list) and to reflect diversity within each category. For example, there were three spaces allocated to fresh pizza in the UKVS. The available products ranged in price from £0.55 to £4.50, varieties included thin base and deep base, and most pizzas had meat-based toppings. The final products selected were a supermarket value-brand cheese and tomato 7-inch pizza (top seller), Pizza Express American pepperoni pizza (one of the top-selling thin base options), and a supermarket own-brand deep pan ham and pineapple pizza (one of the top-selling deep pan options). *Meal Deal* products that were not available online were selected from actual products available in a real small store. A total of 530 real products were selected, which is similar to the original Dutch version of the virtual supermarket, containing 512 products [22]. Full details on the selected products are available from the authors on request.

Moreover, 3D models of the selected UK products were created in Blender (Blender Foundation, Amsterdam, Netherlands), an open source product modeling software [25], using images provided by Brandbank (one of the largest providers of digital product information [26]). The 3D models were designed to replicate the real products (eg, branding, size, color, and style of packaging). Where the real products were supermarket own-brand varieties, the supermarket name was blurred in the 3D model, but all other aspects of the packing were retained. Brand names (eg, Heinz) were retained in the 3D models. Nutritional information was provided by Brandbank and supplemented by matching products with online equivalents. Usual prices (ie, excluding offers) for the selected products were collected from the same supermarket website in January 2016.

### Software Testing

Following the development of the UKVS, software testing was conducted with a convenience sample of 20 adults to ensure the software was working appropriately. Software testers completed the same study procedure that was used in this study and were then interviewed in person or over the phone. The protocol and detailed results from software testing are available from the authors on request. Minor changes were made to the content and layout of participant information screens (eg, information on expected time commitment and additions to the frequently asked questions section) based on software testing participants’ comments.

### Participants

For the actual study, potential participants had to be older than 18 years, able to speak and read English, be the primary household shopper, have access to a computer with a working Internet connection, have an email address, and be confident in using basic computer skills. As data collection in the UKVS is conducted at the household level, only one person from any household was eligible to take part.

### Setting

Participants could complete the study remotely from any location with access to a computer and Internet connection. Participants were recruited, consented, and completed the study online, and data were transferred securely to a university-hosted server via the Internet.

### Sample Size

As this was a feasibility study, no formal sample size calculations were conducted. This feasibility study aimed to recruit 30 participants in each UK equivalized income tertile. The cut-offs for equivalized income tertiles were derived from the Living Costs and Food Survey [23]: low income was defined as equivalized income <£12,844 per year, middle income was £12,844 to £21,372 per year, and high income was >£21,372 per year. On the basis of dropout rates of around 25% in previous virtual supermarket studies [11-13], it was anticipated that recruiting 30 participants in each income tertile would result in approximately 23 study completers per income tertile.

### Recruitment

Participants were recruited via a combination of a free Web-based participant recruitment website [27], Facebook adverts, and snowballing. Recruitment took place over 8 weeks beginning March 2016. The Call for Participants advert was displayed for the entirety of the recruitment period. Facebook adverts were planned for the first 30 days, with a maximum lifetime budget of £250. On the basis of recruitment from previous studies using Facebook adverts [2-5], we estimated that the adverts would generate an average of 58 clicks per day and lead to daily recruitment of 3.6 participants. Additional strategies were in place to recruit through community groups if the estimated sample size was not met in the first 30 days of recruitment.

### Procedure

Participants read the participant information sheet and completed a Web-based consent form on the UKVS website. Upon submission of the consent form, the participant received an automated email with a unique participant identifier or password combination and a link to download the UKVS software. Participants were sent email reminders 1 and 2 weeks after consent if they had not completed the study procedure. Email reminders have previously been shown to increase response rates, but it has been suggested that more than two reminders increase the number of people who view the email as spam [28].

The UKVS study procedure consisted of a preshop questionnaire that gathered sociodemographic details and shopping habits of the household, two *next-day* shopping tasks, and a postshop questionnaire that gathered participant responses to the UKVS software. The participants completed the entire study procedure in one sitting. For the shopping tasks, participants were provided with the following instructions: “Imagine that you have no food or drink in the house (apart from herbs and spices). It is the evening and you are going to the supermarket to buy all the food and drinks for your household for tomorrow. You only need to buy the foods that you would normally purchase in the shop. For example, if you have lunch in the canteen at work, you don’t need to buy lunch in the UK Virtual Supermarket.” We refer to this shopping task as a next-day shopping task throughout the paper, as it requires participants to choose enough food for at least the next day. No restrictions were placed on the total amount that participants could purchase; we expected purchases to be in excess of food requirements for the next day owing to package size restriction (eg, breakfast cereal box is likely to last more than 1 day). Participants were told to imagine that the second shopping task took place a week after the first shopping task. This procedure is similar to instructions that have been provided to participants in other studies examining responses to food price changes [14-16]. All purchases were virtual—participants did not use their own money, and they did not receive actual food products purchased in the UKVS.

### Outcome Measures and Analysis

Outcome measures were collected in relation to four domains: recruitment, participant characteristics, participant purchases, and participant feedback on the UKVS. Participant characteristics collected included age, gender, household income, occupation, and typical shopping habits (eg, usual spend, usual supermarket, and proportion of food purchased in supermarkets). Expenditure and quantity data for participant purchases in the UKVS were collected. UKVS purchase data were combined with each products’ nutritional information to determine the total nutrient content of the basket for energy, protein, carbohydrates, sugars, total fat, saturated fat, salt, and fiber. We also calculated the percentage of the sample that made purchases in each food category. Participants’ feedback was gathered in relation to ease and understanding of the shopping tasks, UKVS product choice, and whether UKVS purchases were representative of typical food shopping behavior.

Using the purchase data, we estimated the number of participants that would be required to detect 5%, 10%, and 20% changes in nutrient purchases using analysis of covariance (ANCOVA) methods in a full trial in the UKVS. We used the power twomeans command for estimating sample size in STATA [29], assuming power=0.8 and Cronbach alpha=.05. These values were then adjusted to the sample size that would be required for ANCOVA using the Borm and colleagues’ method that incorporated estimates of the correlation between the two shops for the nutritional variables [30].

### Ethical Approval

The feasibility study received ethical approval from the University of Oxford Medical Sciences Inter-Divisional Research Ethics Committee (reference no. MSD-IDREC-C1-2013-149).

## Results

### Recruitment

**Figure 2:**
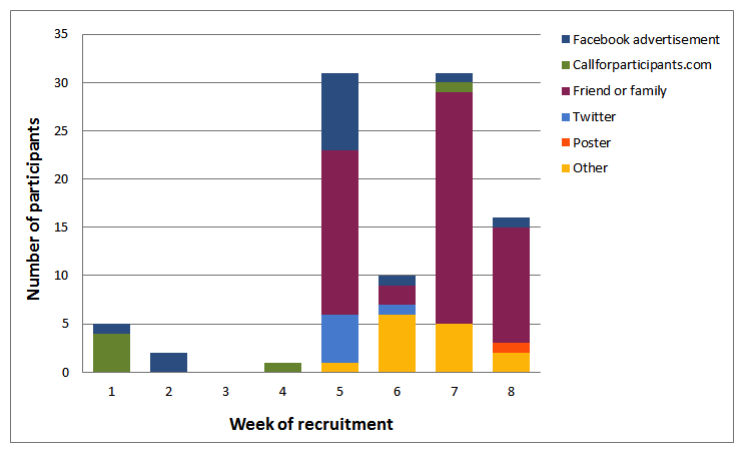
Recruitment over time, by recruitment method.

A total of 96 participants consented to take part in the feasibility study. Figure 2 shows the number of participants recruited in each week of the study by recruitment method. No participants were recruited in week 3 because the Facebook adverts were temporarily suspended to review the study website.

### Differences in Recruitment by Equivalized Income

A total of 30% (29/96) of participants were classified as belonging to the lowest (national) income tertile, 16% (16/96) to the middle income tertile, and 26% (25/96) to the highest income tertile. Furthermore, 27% (26/96) of the participants did not provide sufficient details for their equivalized household income to be calculated. Further details of recruitment method by income tertile are provided in the Multimedia Appendix 1.

### Facebook Adverts

Facebook estimated that there were 5.4 million users daily that met the advert target audience. Demographic characteristics for those who saw and clicked on the adverts are shown in the Multimedia Appendix 1. In total, the Facebook adverts were shown on 374,996 occasions to 183,399 Facebook users. The adverts generated 690 clicks through to the UKVS study website.

### Completion and Participant Characteristics

Out of the 96 participants, 46 fully completed the study procedure, and 2 participants partially completed it (only one shopping task completed). There were significant differences in completion by household size, income, and equivalized income tertile, with lower completion rates in smaller and poorer households. Demographic characteristics for completers and noncompleters are shown in Table 1, with further demographic details for completers presented in the Multimedia Appendix 1.

### Participant Feedback

Table 2 displays participant responses to statements relating to the ease of use, product choice, and similarity of UKVS purchases to real purchases. The majority of participants appeared to have adhered to the instructions for the shopping tasks. Typical weekly budget correlated with the amount spent in shopping tasks (*r*=.56). The concept of a next-day shopping task appeared familiar to most participants. Furthermore, 24 (50%, 24/48) participants reported that they do next-day shopping tasks monthly or more often, and 10 (22%, 10/48) participants reported doing next-day shopping tasks at least a few times per year. However, 7 (15%, 7/48) participants reported that they never did next-day shopping tasks.

**Table 1:** Characteristics of completers and noncompleters.

Characteristics		Completers (n=48)	Noncompleters (n=48)	Total (n=96)	*P* value^a^
Age in years, mean (standard deviation)		38.5 (2.3)	37.3 (2.3)	37.9 (1.6)	.69
**Household size, n (%)**					.03^b^
	1	16 (33)	12 (25)	28 (29)	
	2	23 (48)	17 (35)	40 (42)	
	3	6 (13)	5 (10)	11 (12)	
	≥4	3 (6)	14 (29)	17 (18)	
**Responsibility for food shopping, n (%)**					.42
	All	26 (54)	28 (58)	54 (56)	
	Most	13 (27)	7 (15)	20 (21)	
	Half	8 (17)	9 (19)	17 (18)	
	Little	1 (2)	3 (6)	4 (4)	
	None	0 (0)	1 (2)	1 (1)	
**Computer type, n (%)**					.68
	Windows 8	8 (17)	7 (15)	15 (16)	
	Windows 7	17 (35)	12 (25)	29 (30)	
	Windows Vista	0 (0)	2 (4)	2 (2)	
	Mac OS	13 (27)	12 (25)	25 (26)	
	Other or unknown	11 (23)	14 (29)	25 (26)	
**Computer age in years, n (%)**					.91
	<1	7 (15)	7 (15)	14 (15)	
	1-5	33 (69)	33 (69)	66 (69)	
	>5	6 (13)	7 (15)	13 (14)	
	Unknown	1 (2)	1 (2)	2 (2)	
**Recruitment method, n (%)**					.14
	Call for participants	3 (6)	3 (6)	6 (6)	
	Facebook advert	4 (8)	10 (21)	14 (15)	
	Friend or family	32 (67)	23 (48)	55 (57)	
	Other	9 (19)	12 (25)	21 (22)	
**Income, n (%)**					.02^b^
	£0-£15,000	9 (19)	11 (23)	20 (21)	
	£15,000-£25,000	14 (29)	4 (8)	18 (19)	
	£25,000-£50,000	13 (27)	9 (19)	22 (23)	
	More than £50,000	7 (15)	9 (19)	16 (17)	
	Unknown	5 (10)	15 (31)	20 (21)	
**Equivalized income tertile, n (%)**					<.01^b^
	Low	8 (17)	21 (44)	29 (30)	
	Middle	16 (33)	0 (0)	16 (16)	
	High	21 (44)	4 (8)	25 (26)	
	Unknown	3 (6)	23 (48)	26 (27)	

^a^Values represent *P* values for Fisher exact test, apart from for age where a *t* test was conducted to test for differences between completers and noncompleters.

^b^Statistically significant difference between completers and noncompleters at *P*<.05.

**Table 2:** Participant perceptions of the United Kingdom Virtual Supermarket (UKVS); n=46.

Statement	Strongly agree or agree, n (%)	Neither agree nor disagree, n (%)	Disagree or strongly disagree, n (%)
The virtual supermarket program was easy to understand	38 (83)	6 (13)	2 (4)
The products I purchased in the virtual supermarket resemble my usual food purchases	41 (89)	5 (11)	0 (0)
I could find my way around the virtual supermarket easily	42 (91)	3 (7)	1 (2)
The virtual supermarket contained sufficient product variety	17 (37)	13 (28)	16 (35)
I felt I had sufficient product choice options in the virtual supermarket	18 (39)	10 (22)	18 (39)
Stock in the virtual supermarket is representative of stock in an actual supermarket	31 (67)	6 (13)	9 (20)
I could find the products I wanted to find in the virtual supermarket relatively easily	36 (78)	8 (17)	2 (4)
I could imagine doing my real-life shopping in the virtual supermarket	21 (46)	8 (17)	17 (37)
Prices in the virtual supermarket are similar to prices in an actual supermarket	26 (57)	14 (30)	6 (13)
In the shopping tasks, I think I spent around the same amount of money in the virtual supermarket as I would have in the same task in real life	27 (59)	12 (26)	7 (15)
In the shopping tasks, I bought the same sorts of food and drink as I would have in the same task in real life	41 (89)	4 (9)	1 (2)

**Table 3:** Food category purchases in the United Kingdom Virtual Supermarket (UKVS).

Food category	Participants that were purchasers, %		Amount spent (£)		Grams purchased	
	Shop 1 (n=48)	Shop 2 (n=46)	Mean^a^ (SD^b^)	Mean difference (SD)^c^	Mean^a^ (SD)	Mean difference (SD)^c^
Bread and cereal products	96	93	4.88 (3.31)	1.22 (3.30)	2056 (1190)	457 (1507)
Fruits and vegetables	96	96	5.24 (3.31)	0.72 (3.02)	2696 (1790)	217 (1626)
Meat and fish	75	83	5.28 (3.30)	−0.11 (4.58)	765 (485)	−47 (777)
Milk and dairy	90	83	4.71 (3.33)	1.12 (2.61)	2061 (1444)	390 (911)
Sugar products	33	37	1.54 (1.18)	−0.14 (0.92)	370 (432)	−67 (336)
Beverages	85	67	7.66 (13.61)	2.47 (9.56)	2418 (5217)	345 (1150)
Composite foods or miscellaneous	83	76	3.83 (2.79)	0.51 (3.03)	746 (503)	129 (655)
Total			29.53 (19.55)	5.80 (13.90)	10,123 (6743)	1424 (4091)

^a^Values represent means for the participants that made purchases in the category.

^b^SD: standard deviation.

^c^First shop minus second shop.

### Variability in Purchases

Details of food category level purchases in the UKVS are shown in Table 3. Participants spent an average of £29.53 per shop (standard deviation [SD] 19.55). On average, participants spent £5.80 (SD 13.90) less in the second shop than the first shop. Average spend was highest for *beverages* and lowest for *sugar products*. Nearly all participants purchased products in *fruits and vegetables* and *bread and cereal products*. Table 4 displays overall nutrient content of purchases in the UKVS; differences between the two shopping tasks are provided to give an indication of within-participant variability. There was considerable variation in the mean nutrients purchased in the UKVS, and this was apparent in all three income groups. The high variability indicates that large sample sizes would be required to detect changes in nutrient purchases in a full trial in the UKVS. The total sample sizes that would be required to detect 5%, 10%, and 20% changes in nutrient purchases are given in the Multimedia Appendix 1.

**Table 4:** Nutrient quantities purchased in the United Kingdom Virtual Supermarket (UKVS) across all completers.

Nutrients		All (n=46)	Lowest income (n=8)	Middle income (n=15)	High income (n=20)
**Mean amounts purchased^a^(SD^b^)**					
	Energy (kcal)	14,479 (8742)	10,247 (5489)	16,354 (8859)	14,282 (9878)
	Protein (g)	836 (969)	1087 (1996)	941 (792)	632 (442)
	Fat (g)	541 (353)	334 (240)	643 (335)	533 (403)
	Saturated fat (g)	194 (125)	117 (91)	235 (130)	183 (129)
	Carbohydrate (g)	1706 (1140)	1155 (547)	1830 (1195)	1764 (1275)
	Sugar (g)	665 (549)	592 (714)	684 (491)	645 (545)
	Fiber (g)	214 (153)	203 (208)	238 (174)	195 (128)
	Sodium (mg)	13,750 (8290)	9634 (4545)	15,452 (9594)	13,829 (8607)
**Mean percentage energy from selected macronutrients^c^(SD)**					
	Protein	22.4 (17.6)	32.5 (39.8)	20.5 (7.5)	19.6 (8.2)
	Fat	33.0 (9.9)	29.2 (11.9)	36.8 (10.5)	31.6 (8.6)
	Saturated fat	11.8 (4.3)	10.8 (5.61)	12.9 (4.7)	11.1 (3.6)
	Carbohydrate	47.2 (9.4)	47.0 (14.1)	43.4 (7.8)	49.9 (8.1)
	Sugar	18.7 (9.1)	22.0 (16.1)	15.7 (5.0)	19.7 (8.1)
	Fiber	3.0 (1.1)	3.7 (2.02)	2.8 (0.9)	2.9 (0.5)
**Mean difference between the two shops^b^(SD)**					
	Kcal	1825 (6732)	−238 (2728)	2437 (6927)	1894 (7884)
	Protein (g)	53 (855)	−497 (1322)	220 (1006)	141 (401)
	Fat (g)	62 (279)	−51 (126)	36 (271)	86 (289)
	Saturated fat (g)	15 (123)	−19 (44)	−10 (113)	25 (119)
	Carbohydrate (g)	259 (1192)	242 (569)	469 (1434)	133 (1286)
	Sugar (g)	63 (409)	101 (272)	163 (421)	−26 (466)
	Fiber (g)	27 (113)	−46 (144)	43 (126)	45 (89)
	Sodium (mg)	2314 (7475)	−299 (3742)	3973 (10,185)	1560 (5712)

^a^First shop minus second shop.

^b^SD: standard deviation.

^c^Average of the average of two shops across participants.

## Discussion

### Summary

This feasibility study set out to assess recruitment, retention, purchasing variability, and participant responses to the newly developed UKVS and to examine differences in the above by household income. We found that completion rates in the UKVS were lower than anticipated but that feedback from participants was positive and similar across all income groups. The results from this study suggest the UKVS would be a feasible tool for examining purchasing behavior in different income groups.

### Comparison With Other Literature

To our best knowledge, the UKVS is the first 3D simulation of a supermarket that has been developed exclusively for research purposes in the United Kingdom. Recent comparisons have shown that virtual reality better represents purchasing behavior in actual brick-and-mortar stores than picture-based approaches [31]. This suggests that the UKVS may elicit more realistic purchasing behavior than other experimental settings, though future direct comparisons between purchases in experimental environments (including the UKVS) and the real-life environments they are designed to replicate (in this case brick-and-mortar stores) are warranted to examine this explicitly.

We are aware that other, non-3D online shopping platforms that allow participants to select from a list of possible food items have been developed in the United Kingdom [30]. Forwood and colleagues’ online shopping platform differs from the UKVS, as it was designed to mimic an online supermarket website rather than a brick-and-mortar store. Online shopping is growing in popularity in the United Kingdom, though the market share remains low at 6% [32]. Given the variability in real food shopping environments, complementary evidence examining different types of purchases (eg, online vs brick-and-mortar stores) is needed to build a complete picture of the likely impacts of specific interventions on purchases.

The product selection in the UKVS is representative for what can be found in a real supermarket, and the tool contains over 500 different products. Other studies using supermarket models to study the impact of HRFTS on food purchases have offered a selection of as few as 60 products [33], though more recent studies have had a selection of as many as 708 products [14,34]. The stores surveyed as part of the UKVS product selection process contained between 2600 and 3300 food products, and online supermarkets contain around 11,000 food products [30]. Smaller product selections in experimental environments may still elicit typical purchasing decisions given the large numbers of similar products in real stores, provided that the most commonly consumed products are represented. For example, there were more than 110 varieties of baked beans available in the online store used for this study.

The UKVS is similar to other virtual supermarkets developed by the same company. The New Zealand version has been validated against real purchases [13], and the Dutch Virtual Supermarket has been used for a number of trials of pricing interventions [11,12,35,36]. Across all the virtual supermarket studies, feedback from participants has been positive. The validation and use of previous virtual supermarkets and positive participant experiences suggest that the UKVS is a good experimental environment for testing the effects of pricing interventions.

This is the first study to examine how suitable the virtual supermarket environment is for examining purchases across different income groups. Positive feedback from study completers suggests that the UKVS is suitable for examining differences in purchases across different income groups. In real supermarkets, different availability of certain foods may influence observed differences in purchasing behavior across different groups. For example, an Australian study found that there are more energy-dense snack foods and soft drinks available in supermarkets in more disadvantaged neighborhoods than in less disadvantaged neighborhoods [37]. As all participants are exposed to the same environment, the UKVS has the potential to examine the contribution of income and other socioeconomic factors to differences in purchasing behavior independent of differences in access and availability.

### Strengths and Limitations of the UK Virtual Supermarket

The use of the existing Dutch Virtual Supermarket as a template for the UKVS considerably reduced the resources required for development. The similarity of the Dutch layout with the layout of surveyed UK stores suggests that using a template from a different country is unlikely to have detracted from the realism of the UKVS.

Although the UKVS was developed primarily to assess the impact of HRFTS, the tool can also be used to assess other supermarket-based interventions. The UKVS software incorporates the ability to provide traffic light labels when participants hover over products, and shelf tags can be added to indicate promotions on a particular product. The UKVS is not designed to test the impact of changing product placement, but this feature could be added in future versions.

The next-day shopping task used in this feasibility study was selected to represent an important aspect of household food purchasing—smaller or top-up purchasing. UK data suggest that an increasing proportion of grocery spend is because of top-up shops compared with main shopping trips. Currently, top-up shops represent 61% of spending [17]. In this study, 50% of participants stated that they would conduct a similar shop to the UKVS task on at least a monthly basis. This suggests that although not comparable to participants’ usual supermarket routines, the task was nevertheless familiar to participants. Similar shopping tasks have been used in previous studies looking at the impact of price on purchases [14-16]. The size and type of shopping task that can be conducted in the UKVS is limited by the experimental environment—nonsupermarket and occasional impulse purchases are not captured. This means that results from trials in the UKVS will need to be combined with trials in other settings to build a full picture of the impact of changing prices on household purchases.

The external validity of UKVS and similar experimental studies is limited by participants not making real purchases [13,15]. Self-report data from this feasibility study suggest that participant purchases were similar to their usual purchases. These responses, coupled with results from the NZVS validation study [13], provide an initial indication that results from trials of pricing interventions in the UKVS would be externally valid. However, continued validation of experimental purchases compared with actual purchases and consumption patterns should be built into future studies of this kind.

### Strengths and Limitations of the Feasibility Study

Completion rate of the study procedure was lower than we had anticipated. In addition, many of the reasons for noncompletion were not known. Completion in previous virtual supermarket studies was around 80% [11,12,35,38], with 60% completion observed in the NZVS validation study where participants had to complete a series of shopping tasks over a 3-week period [13]. Difficulties downloading the software and incorrect entry of email address appeared to contribute to noncompletion in this study. In the future, this could be minimized with additional methods to ensure participants receive user details (eg, text message [short service message, SMS] with user identifier or password, in addition to email and multiple email address entry).

There were several aspects of the feasibility study process that could be improved for future studies. Unfortunately, we were not able to collect data on the number of noncompleters who attempted to download the software. In addition, the registration process could be improved to better screen participants; one person who registered did not meet the criterion of being a primary household shopper, and it may have been possible for multiple people from the same household to enroll without our knowledge.

The relationship of completion with household income and participant feedback on problems with the download procedure indicate that some participants may need more support to take part in the study. In Great Britain, 82% of adults use the Internet every day or almost every day, and 89% of households have an Internet connection [39]. Of the 11% of households in Great Britain with no Internet access, 59% report that this was because they did not need Internet access, 21% reported that this was because of lack of skills, and 18% reported cost barriers [39]. These data suggest that some selection bias may have been created because of computer availability, but the magnitude of this bias is likely to be small. The sample in this study had high levels of education; 85.4% of participants had degree level or above education compared with 27.2% of adults in England and Wales [40]. To improve recruitment and completion across all socioeconomic groups, future studies could adopt mixed recruitment approaches where participants have the option of remote participation or completing the study procedure at set locations where both computers and assistance from researchers are available to overcome barriers related to skill and computer cost. Completers across all three income tertiles appeared to have similar responses to the UKVS; this suggests that if completion rates were improved, the UKVS would be suitable for examining the impacts of interventions across different income groups.

We found that Facebook was less successful as a recruitment strategy than anticipated from previous literature [2-5]. In this study, we have provided details on views and clicks generated via Facebook to enable future comparisons of recruitment rates across different study types. In the PriceExaM study that was recruiting in the same time period using the NZVS [41], Facebook adverts were more successful than observed in this study, but full analysis is still underway (Wilma Waterlander and Rita George, personal communication). The content of the UKVS adverts was similar to that in PriceExaM; features that differed were that PriceExaM adverts contained a video, and incentives and duration differed across the two studies (NZ $40 payment for completing 5 shopping tasks over 5 weeks vs prize draw for completing two immediate shopping tasks). Future UKVS studies could consider testing a guaranteed incentive structure and incorporating videos into adverts to see whether these improve recruitment and retention in the United Kingdom.

This feasibility study collected purchasing data across a broad range of outcome measures to establish sample sizes required to detect changes across multiple outcome measures. Patterns of purchasing behavior in the UKVS reveal the types of intervention that are more or less suited to being examined in the UKVS environment. The UKVS would be an appropriate environment to examine the impacts of interventions that target a broad spectrum of foods, as the majority of completers made purchases across the majority of categories. However, the UKVS is less suited to trials targeting more specific food categories. For example, only one-third of participants made purchases in the *sugar products* category, which included chocolates and confectionery. This means that the impact of price changes on chocolates and confectionery would be estimated with poor precision in UKVS studies.

### Suggestions for Future Research

RCTs in the UKVS could provide valuable evidence of the potential effectiveness of HRFTS in the United Kingdom. However, as the UKVS represents a single purchasing environment, it is important that data from the UKVS are combined with information from other settings (eg, canteens, vending machines, fast food vendors, and restaurants). Schroeter et al [42] note that a tax on away-from-home foods could result in overall increases in food consumption because of substitution behavior. Ideally, we need studies that can assess overall changes in purchases across multiple settings to establish the overall impacts of HRFTS on purchases.

Resource constraints are likely to continue to be an important barrier to testing pricing interventions in real life; artificial environments such as the UKVS can help fill this gap. Continued research is required to improve the external validity of experimental studies by identifying features of trial design that prompt realistic purchasing behavior in experimental environments. For example, Epstein et al [43] charged participants for purchases made in an experimental setting from the (large) monetary incentive that was provided. They found that participants still spent more than they would in real life, possibly because of the additional income afforded by the incentive. An alternative approach may be to offer decoupled incentives. Households allocate budgets to particular categories of expenditure, and people are reluctant to spend money in one budget on items that fall under another budget [44,45]. By providing incentives in a different form (eg, vouchers for clothing or payment of energy bills), experiments may prompt more realistic food purchasing behavior and provide adequate financial compensation to participants.

### Conclusions

Participant feedback on the UKVS was positive, and self-report data suggest that the UKVS did reflect participants’ real purchasing decisions. However, this study revealed important limitations with recruitment and retention in the UKVS that need to be addressed before the software can be used for a full trial. The results of this study suggest that the UKVS would be a feasible tool for examining purchasing behavior in different income groups if these issues surrounding recruitment were resolved (eg, by providing participants the option to participate at study centers in addition to online).

## Appendix

Multimedia Appendix 1 Supplementary information.

## Abbreviations

3D: three-dimensional
ANCOVA: analysis of covariance
HRFTS: health-related food taxes and subsidies
NZVS: New Zealand Virtual Supermarket
RCT: randomized controlled trial
SD: standard deviation
UKVS: United Kingdom Virtual Supermarket

## References

[1] Forouzanfar Mohammad H, Alexander Lily, Anderson H Ross, Bachman Victoria F, Biryukov Stan, Brauer Michael, Burnett Richard, Casey Daniel, Coates Matthew M, Cohen Aaron, Delwiche Kristen, Estep Kara, Frostad Joseph J, Astha K C, Kyu Hmwe H, Moradi-Lakeh Maziar, Ng Marie, Slepak Erica Leigh, Thomas Bernadette A, Wagner Joseph, Aasvang Gunn Marit, Abbafati Cristiana, Abbasoglu Ozgoren Ayse, Abd-Allah Foad, Abera Semaw F, Aboyans Victor, Abraham JP, Abraham Jerry Puthenpurakal, Abubakar Ibrahim, Abu-Rmeileh Niveen M E, Aburto Tania C, Achoki Tom, Adelekan Ademola, Adofo Koranteng, Adou Arsène K, Adsuar José C, Afshin Ashkan, Agardh Emilie E, Al Khabouri Mazin J, Al Lami Faris H, Alam Sayed Saidul, Alasfoor Deena, Albittar Mohammed I, Alegretti Miguel A, Aleman Alicia V, Alemu Zewdie A, Alfonso-Cristancho Rafael, Alhabib Samia, Ali MK, Ali Mohammed K, Alla François, Allebeck Peter, Allen Peter J, Alsharif Ubai, Alvarez Elena, Alvis-Guzman Nelson, Amankwaa Adansi A, Amare Azmeraw T, Ameh Emmanuel A, Ameli Omid, Amini Heresh, Ammar Walid, Anderson Benjamin O, Antonio Carl Abelardo T, Anwari Palwasha, Argeseanu Cunningham Solveig, Arnlöv Johan, Arsenijevic Valentina S Arsic, Artaman Al, Asghar Rana J, Assadi Reza, Atkins Lydia S, Atkinson Charles, Avila Marco A, Awuah Baffour, Badawi Alaa, Bahit Maria C, Bakfalouni Talal, Balakrishnan Kalpana, Balalla Shivanthi, Balu Ravi Kumar, Banerjee Amitava, Barber Ryan M, Barker-Collo Suzanne L, Barquera Simon, Barregard Lars, Barrero Lope H, Barrientos-Gutierrez Tonatiuh, Basto-Abreu Ana C, Basu S, Basu Sanjay, Basulaiman Mohammed O, Batis Ruvalcaba Carolina, Beardsley Justin, Bedi Neeraj, Bekele Tolesa, Bell Michelle L, Benjet Corina, Bennett Derrick A, Benzian Habib, Bernabé Eduardo, Beyene Tariku J, Bhala Neeraj, Bhalla Ashish, Bhutta Zulfiqar A, Bikbov Boris, Bin Abdulhak Aref A, Blore Jed D, Blyth Fiona M, Bohensky Megan A, Bora Başara Berrak, Borges Guilherme, Bornstein Natan M, Bose Dipan, Boufous Soufiane, Bourne Rupert R, Brainin Michael, Brazinova Alexandra, Breitborde Nicholas J, Brenner Hermann, Briggs Adam D M, Broday David M, Brooks Peter M, Bruce Nigel G, Brugha Traolach S, Brunekreef Bert, Buchbinder Rachelle, Bui Linh N, Bukhman Gene, Bulloch Andrew G, Burch Michael, Burney Peter G J, Campos-Nonato Ismael R, Campuzano Julio C, Cantoral Alejandra J, Caravanos Jack, Cárdenas Rosario, Cardis Elisabeth, Carpenter David O, Caso Valeria, Castañeda-Orjuela Carlos A, Castro Ruben E, Catalá-López Ferrán, Cavalleri Fiorella, Çavlin Alanur, Chadha Vineet K, Chang Jung-Chen, Charlson Fiona J, Chen W, Chen Z, Chen Zhengming, Chiang Peggy P, Chimed-Ochir Odgerel, Chowdhury Rajiv, Christophi Costas A, Chuang Ting-Wu, Chugh Sumeet S, Cirillo Massimo, Claßen Thomas K D, Colistro Valentina, Colomar Mercedes, Colquhoun Samantha M, Contreras Alejandra G, Cooper Cyrus, Cooperrider Kimberly, Cooper Leslie T, Coresh Josef, Courville Karen J, Criqui Michael H, Cuevas-Nasu Lucia, Damsere-Derry James, Danawi Hadi, Dandona R, Dandona Rakhi, Dargan Paul I, Davis Adrian, Davitoiu Dragos V, Dayama Anand, de Castro E Filipa, De la Cruz-Góngora Vanessa, De Leo Diego, de Lima Graça, Degenhardt Louisa, del Pozo-Cruz Borja, Dellavalle Robert P, Deribe Kebede, Derrett Sarah, Des Jarlais Don C, Dessalegn Muluken, deVeber Gabrielle A, Devries Karen M, Dharmaratne Samath D, Dherani Mukesh K, Dicker Daniel, Ding Eric L, Dokova Klara, Dorsey E Ray, Driscoll Tim R, Duan Leilei, Durrani Adnan M, Ebel Beth E, Ellenbogen Richard G, Elshrek Yousef M, Endres Matthias, Ermakov Sergey P, Erskine Holly E, Eshrati Babak, Esteghamati Alireza, Fahimi Saman, Faraon Emerito Jose A, Farzadfar Farshad, Fay Derek F J, Feigin Valery L, Feigl Andrea B, Fereshtehnejad Seyed-Mohammad, Ferrari Alize J, Ferri Cleusa P, Flaxman Abraham D, Fleming Thomas D, Foigt Nataliya, Foreman Kyle J, Paleo Urbano Fra, Franklin Richard C, Gabbe Belinda, Gaffikin Lynne, Gakidou Emmanuela, Gamkrelidze Amiran, Gankpé Fortuné G, Gansevoort Ron T, García-Guerra Francisco A, Gasana Evariste, Geleijnse Johanna M, Gessner Bradford D, Gething Pete, Gibney Katherine B, Gillum Richard F, Ginawi Ibrahim A M, Giroud Maurice, Giussani Giorgia, Goenka Shifalika, Goginashvili Ketevan, Gomez Dantes Hector, Gona Philimon, Gonzalez de Cosio Teresita, González-Castell Dinorah, Gotay Carolyn C, Goto Atsushi, Gouda Hebe N, Guerrant Richard L, Gugnani Harish C, Guillemin Francis, Gunnell David, Gupta R, Gupta Rajeev, Gutiérrez Reyna A, Hafezi-Nejad Nima, Hagan Holly, Hagstromer Maria, Halasa Yara A, Hamadeh Randah R, Hammami Mouhanad, Hankey Graeme J, Hao Yuantao, Harb Hilda L, Haregu Tilahun Nigatu, Haro Josep Maria, Havmoeller Rasmus, Hay Simon I, Hedayati Mohammad T, Heredia-Pi Ileana B, Hernandez Lucia, Heuton Kyle R, Heydarpour Pouria, Hijar Martha, Hoek Hans W, Hoffman Howard J, Hornberger John C, Hosgood H Dean, Hoy Damian G, Hsairi Mohamed, Hu H, Hu Howard, Huang JJ, Huang John J, Hubbell Bryan J, Huiart Laetitia, Husseini Abdullatif, Iannarone Marissa L, Iburg Kim M, Idrisov Bulat T, Ikeda Nayu, Innos Kaire, Inoue Manami, Islami Farhad, Ismayilova Samaya, Jacobsen Kathryn H, Jansen Henrica A, Jarvis Deborah L, Jassal Simerjot K, Jauregui Alejandra, Jayaraman Sudha, Jeemon Panniyammakal, Jensen Paul N, Jha Vivekanand, Jiang G, Jiang Y, Jiang Ying, Jonas Jost B, Juel Knud, Kan Haidong, Kany Roseline Sidibe S, Karam Nadim E, Karch André, Karema Corine K, Karthikeyan Ganesan, Kaul Anil, Kawakami Norito, Kazi Dhruv S, Kemp Andrew H, Kengne Andre P, Keren Andre, Khader Yousef S, Khalifa Shams Eldin Ali Hassan, Khan Ejaz A, Khang Young-Ho, Khatibzadeh Shahab, Khonelidze Irma, Kieling Christian, Kim S, Kim Y, Kim Yunjin, Kimokoti Ruth W, Kinfu Yohannes, Kinge Jonas M, Kissela Brett M, Kivipelto Miia, Knibbs Luke D, Knudsen Ann Kristin, Kokubo Yoshihiro, Kose M Rifat, Kosen Soewarta, Kraemer Alexander, Kravchenko Michael, Krishnaswami Sanjay, Kromhout Hans, Ku Tiffany, Kuate Defo Barthelemy, Kucuk Bicer Burcu, Kuipers Ernst J, Kulkarni VS, Kulkarni Veena S, Kumar G Anil, Kwan Gene F, Lai Taavi, Lakshmana Balaji Arjun, Lalloo Ratilal, Lallukka Tea, Lam Hilton, Lan Qing, Lansingh Van C, Larson Heidi J, Larsson Anders, Laryea Dennis O, Lavados Pablo M, Lawrynowicz Alicia E, Leasher Janet L, Lee Jong-Tae, Leigh James, Leung Ricky, Levi Miriam, Li Y, Li Yongmei, Liang X, Liang Xiaofeng, Lim Stephen S, Lindsay M Patrice, Lipshultz Steven E, Liu Y, Liu Yang, Lloyd Belinda K, Logroscino Giancarlo, London Stephanie J, Lopez Nancy, Lortet-Tieulent Joannie, Lotufo Paulo A, Lozano Rafael, Lunevicius Raimundas, Ma S, Ma Stefan, Machado Vasco M P, MacIntyre Michael F, Magis-Rodriguez Carlos, Mahdi Abbas A, Majdan Marek, Malekzadeh Reza, Mangalam Srikanth, Mapoma Christopher C, Marape Marape, Marcenes Wagner, Margolis David J, Margono Christopher, Marks Guy B, Martin Randall V, Marzan Melvin B, Mashal Mohammad T, Masiye Felix, Mason-Jones Amanda J, Matsushita Kunihiro, Matzopoulos Richard, Mayosi Bongani M, Mazorodze Tasara T, McKay Abigail C, McKee Martin, McLain Abigail, Meaney Peter A, Medina Catalina, Mehndiratta Man Mohan, Mejia-Rodriguez Fabiola, Mekonnen Wubegzier, Melaku Yohannes A, Meltzer Michele, Memish Ziad A, Mendoza Walter, Mensah George A, Meretoja Atte, Mhimbira Francis Apolinary, Micha Renata, Miller Ted R, Mills Edward J, Misganaw Awoke, Mishra Santosh, Mohamed Ibrahim Norlinah, Mohammad Karzan A, Mokdad Ali H, Mola Glen L, Monasta Lorenzo, Montañez Hernandez Julio C, Montico Marcella, Moore Ami R, Morawska Lidia, Mori Rintaro, Moschandreas Joanna, Moturi Wilkister N, Mozaffarian Dariush, Mueller Ulrich O, Mukaigawara Mitsuru, Mullany Erin C, Murthy Kinnari S, Naghavi Mohsen, Nahas Ziad, Naheed Aliya, Naidoo Kovin S, Naldi Luigi, Nand Devina, Nangia Vinay, Narayan K M Venkat, Nash Denis, Neal Bruce, Nejjari Chakib, Neupane Sudan P, Newton Charles R, Ngalesoni Frida N, Ngirabega Jean de Dieu, Nguyen NT, Nguyen Nhung T, Nieuwenhuijsen Mark J, Nisar Muhammad I, Nogueira José R, Nolla Joan M, Nolte Sandra, Norheim Ole F, Norman Rosana E, Norrving Bo, Nyakarahuka Luke, Oh In-Hwan, Ohkubo Takayoshi, Olusanya Bolajoko O, Omer Saad B, Opio John Nelson, Orozco Ricardo, Pagcatipunan Rodolfo S, Pain Amanda W, Pandian Jeyaraj D, Panelo Carlo Irwin A, Papachristou Christina, Park Eun-Kee, Parry Charles D, Paternina Caicedo Angel J, Patten Scott B, Paul Vinod K, Pavlin Boris I, GBD 2013 Risk Factors Collaborators (2015). Global, regional, and national comparative risk assessment of 79 behavioural, environmental and occupational, and metabolic risks or clusters of risks in 188 countries, 1990-2013: a systematic analysis for the Global Burden of Disease Study 2013. Lancet.

[2] (2015). Institute for Health Metrics and Evaluation.

[3] Department for Environment Food and Rural Affairs.

[4] Julia C, Méjean C, Vicari F, Péneau S, Hercberg S (2015). Public perception and characteristics related to acceptance of the sugar-sweetened beverage taxation launched in France in 2012. Public Health Nutr.

[5] Colchero MA, Popkin BM, Rivera JA, Ng SW (2016). Beverage purchases from stores in Mexico under the excise tax on sugar sweetened beverages: observational study. Br Med J.

[6] (2015). World Cancer Research Fund.

[7] Jones CM (2016). The UK sugar tax - a healthy start?. Br Dent J.

[8] Thow AM, Downs S, Jan S (2014). A systematic review of the effectiveness of food taxes and subsidies to improve diets: understanding the recent evidence. Nutr Rev.

[9] (2010). Global status report on noncommunicable diseases.

[10] (2017). Office for National Statistics.

[11] Waterlander WE, Ni Mhurchu C, Steenhuis IH (2014). Effects of a price increase on purchases of sugar sweetened beverages. Results from a randomized controlled trial. Appetite.

[12] Waterlander WE, Steenhuis IH, de Boer MR, Schuit AJ, Seidell JC (2012). Introducing taxes, subsidies or both: the effects of various food pricing strategies in a web-based supermarket randomized trial. Prev Med.

[13] Waterlander WE, Jiang Y, Steenhuis IH, Ni Mhurchu C (2015). Using a 3D virtual supermarket to measure food purchase behavior: a validation study. J Med Internet Res.

[14] Nederkoorn C, Havermans RC, Giesen JC, Jansen A (2011). High tax on high energy dense foods and its effects on the purchase of calories in a supermarket. An experiment. Appetite.

[15] Darmon N, Lacroix A, Muller L, Ruffieux B (2014). Food price policies improve diet quality while increasing socioeconomic inequalities in nutrition. Int J Behav Nutr Phys Act.

[16] Giesen JC, Havermans RC, Nederkoorn C, Jansen A (2012). Impulsivity in the supermarket. Responses to calorie taxes and subsidies in healthy weight undergraduates. Appetite.

[17] (2014). Kantar Worldpanel.

[18] Mizdrak A, Scarborough P, Waterlander WE, Rayner M (2015). Differential responses to food price changes by personal characteristic: a systematic review of experimental studies. PLoS One.

[19] Thow AM, Jan S, Leeder S, Swinburn B (2010). The effect of fiscal policy on diet, obesity and chronic disease: a systematic review. Bull World Health Organ.

[20] Eyles H, Ni Mhurchu C, Nghiem N, Blakely T (2012). Food pricing strategies, population diets, and non-communicable disease: a systematic review of simulation studies. PLoS Med.

[21] Mhurchu CN, Blakely T, Funaki-Tahifote M, McKerchar C, Wilton J, Chua S, Jiang Y (2009). Inclusion of indigenous and ethnic minority populations in intervention trials: challenges and strategies in a New Zealand supermarket study. J Epidemiol Community Health.

[22] Waterlander WE, Scarpa M, Lentz D, Steenhuis IH (2011). The virtual supermarket: an innovative research tool to study consumer food purchasing behaviour. BMC Public Health.

[23] (2015). Living Costs and Food Survey, 2011-2013: Secure Access. Office for National Statistics, Department for Environment Food and Rural Affairs.

[24] Sainsburys.co.uk.

[25] (2015). Blender.

[26] Brandbank.

[27] Callforparticipants.

[28] Lyons LA, Cude B, Lawrence FC, Gutter M (2005). Conducting research online: challenges facing researchers in family and consumer sciences. Fam Consum Sci Res J.

[29] (2015). StataCorp.

[30] Borm GF, Fransen J, Lemmens WA (2007). A simple sample size formula for analysis of covariance in randomized clinical trials. J Clin Epidemiol.

[31] van Herpen E, van den Broek E, van Trijp HC, Yu T (2016). Can a virtual supermarket bring realism into the lab? Comparing shopping behavior using virtual and pictorial store representations to behavior in a physical store. Appetite.

[32] (2017). Department for Environment Food and Rural Affairs.

[33] Epstein LH, Dearing KK, Paluch RA, Roemmich JN, Cho D (2007). Price and maternal obesity influence purchasing of low- and high-energy-dense foods. Am J Clin Nutr.

[34] Giesen JC, Payne CR, Havermans RC, Jansen A (2011). Exploring how calorie information and taxes on high-calorie foods influence lunch decisions. Am J Clin Nutr.

[35] Waterlander WE, Steenhuis IH, de Boer MR, Schuit AJ, Seidell JC (2012). The effects of a 25% discount on fruits and vegetables: results of a randomized trial in a three-dimensional web-based supermarket. Int J Behav Nutr Phys Act.

[36] Waterlander WE, de Boer MR, Schuit AJ, Seidell JC, Steenhuis IH (2013). Price discounts significantly enhance fruit and vegetable purchases when combined with nutrition education: a randomized controlled supermarket trial. Am J Clin Nutr.

[37] Cameron AJ, Thornton LE, McNaughton SA, Crawford D (2013). Variation in supermarket exposure to energy-dense snack foods by socio-economic position. Public Health Nutr.

[38] Waterlander WE, Steenhuis IH, de Boer MR, Schuit AJ, Seidell JC (2013). Effects of different discount levels on healthy products coupled with a healthy choice label, special offer label or both: results from a web-based supermarket experiment. Int J Behav Nutr Phys Act.

[39] Office for National Statistics.

[40] Office for National Statistics.

[41] Waterlander WE, Blakely T, Nghiem N, Cleghorn CL, Eyles H, Genc M, Wilson N, Jiang Y, Swinburn B, Jacobi L, Michie J, Ni Mhurchu C (2016). Study protocol: combining experimental methods, econometrics and simulation modelling to determine price elasticities for studying food taxes and subsidies (The Price ExaM Study). BMC Public Health.

[42] Schroeter C, Lusk J, Tyner W (2008). Determining the impact of food price and income changes on body weight. J Health Econ.

[43] Epstein LH, Finkelstein E, Raynor H, Nederkoorn C, Fletcher KD, Jankowiak N, Paluch RA (2015). Experimental analysis of the effect of taxes and subsides on calories purchased in an on-line supermarket. Appetite.

[44] Thaler RH (1999). Mental Accounting Matters. J Behav Decis Mak.

[45] Thaler RH (2015). Misbehaving: The Making of Behavioural Economics.

