# Supplementary material for: Using a UK Virtual Supermarket to Examine Purchasing Behavior Across Different Income Groups in the United Kingdom: Development and Feasibility Study
Source: J Med Internet Res. 2017 Oct 9;19(10):e343. doi: 10.2196/jmir.7982 (PMC5653905; doi:10.2196/jmir.7982)
Supplement: Multimedia Appendix 1 [file jmir_v19i10e343_app1.pdf]

i) *Recruitment methods by equivalised income tertile*

	Equivalised income tertile			
	Lowest (n=29)	Middle (n=16)	Highest (n=25)	Unknown (n=26)
Call for participants	1 (3.4%)	1 (6.3%)	2 (8.0%)	2 (7.7%)
Facebook	5 (17.2%)	2 (12.5%)	1 (4.0%)	6 (23.1%)
Friend/family	17 (58.6%)	10 (62.5%)	15 (60.0%)	13 (50.0%)
Other	6 (20.7%)	3 (18.8%)	7 (28%)	5 (19.2%)

ii) *Demographic characteristics of those reached through Facebook*

	Number reached	Number that clicked through to study website
Gender		
- Male	106,182	503
- Female	68,751	163
- Unknown	8,467	24
Age (years)		
- 13-17[a]	32	0
- 18-24	19,479	29
- 25-34	12,763	34
- 35-44	12,095	22
- 45-54	29,506	69
- 55-64	48,826	207
- 65+	60,698	329
Region		
- England	146,382	537
- Wales	11,395	46
- Scotland	20,944	79
- Northern Ireland	4,679	27
Total	183,399	690

[a] We specified that the advert was only to be shown to those aged 18 years or older, yet a small number of under 18s were also shown the advert.

iii) *Further demographic description of completers*

Household income	
- Mean	£34,966
- SE	£4,018
Equivalised household income	
- Mean	£23,937

- SE	£2,380
Equivalised income tertile	
- Low income	8
- Middle income	15
- High income	20
- Unknown	3
Gender	
- Male	14 (29.2%)
- Female	34 (70.8%)
Ethnicity	
- White	43 (89.6%)
- Mixed/multiple	2 (4.2%)
- Asian/Asian British	2 (4.2%)
- Other ethnic group	1 (2.1%)
Education	
- No qualifications	2 (4.2%)
- O levels/CSEs/GCSEs or equivalent	1 (2.1%)
- A levels or equivalent	3 (6.3%)
- Degree	40 (83.3%)
- Higher degree	1 (2.1%)
- Foreign qualifications	1 (2.1%)
Current work status	
- Unemployed	4 (8.3%)
- Full-time employed	18 (37.5%)
- Part-time employed	6 (12.5%)
- Self-employed	5 (10.4%)
- Looking after children/family	1 (2.1%)
- Other	14 (29.2%)
Current occupation	
- Higher managerial and professional	6 (12.5%)
- Lower managerial and professional	19 (39.6%)
- Intermediate	6 (12.5%)
- Routine	1 (2.1%)
- Never worked or long term unemployed	2 (4.2%)
- Other	13 (27.1%)
- Don't know/ Prefer not to say	1 (2.1%)
Mean weekly household grocery budget (£) (SE)	65.10 (4.90)
Proportion of all food purchased in supermarkets	
- All	17 (35.4%)
- Most	26 (54.2%)
- Half	5 (10.4%)
- Little	0 (0.0%)
- None	0 (0.0%)
Supermarket frequency	
- Every day	2 (4.2%)
- Every other day	5 (10.4%)

- 3/4 times per week	11 (22.9%)
- Twice weekly	14 (29.2%)
- Once weekly	12 (25.0%)
- Every other week	4 (8.3%)
Usual grocery routine	
- Big shop	5 (10.4%)
- Big shop with top ups	22 (45.8%)
- Smaller shops	20 (41.7%)
- Daily purchases	1 (2.1%)
Dietary requirements	
- Yes	20 (41.7%)
- No	28 (58.3%)
Usual supermarket	
- Tesco	19 (39.6%)
- Sainsbury's	14 (29.2%)
- Asda	4 (8.3%)
- Waitrose	4 (8.3%)
- Lidl or Aldi	3 (6.3%)
- Other	4 (8.3%)

iv) Sample sizes required to detect nutrient changes in randomised controlled trials using the UK Virtual Supermarket

	Sample size required		
Outcome measure	5% change	10% change	20% change
Fruits and vegetables (g)	2,511	628	158
Energy (kcal)	2,410	605	152
Protein (g)	11,629	2,909	729
Fat (g)	2,844	710	180
Saturated fat (g)	4,420	1,106	278
Carbohydrates (g)	5,623	1,408	354
Sugars (g)	3,819	959	242
Fibre (g)	2,693	675	171
Sodium (mg)	2,083	522	131
